# Skp2 expression unfavorably impacts survival in resectable esophageal squamous cell carcinoma

**DOI:** 10.1186/1479-5876-10-73

**Published:** 2012-05-18

**Authors:** Yi Liang, Xue Hou, Qian Cui, Tie-Bang Kang, Jian-Hua Fu, Lan-Jun Zhang, Rong-Zhen Luo, Jie-Hua He, Yi-Xin Zeng, Hao-Xian Yang

**Affiliations:** 1 State Key Laboratory of Oncology in South China, Guangzhou, 510060, People's Republic of China; 2 Department of Experimental Research, Sun Yat-sen University Cancer Center, Guangzhou, 510060, People's Republic of China; 3 Department of Medical Oncology, Sun Yat-sen University Cancer Center, Guangzhou, 510060, People's Republic of China; 4 Department of Thoracic Surgery, Sun Yat-sen University Cancer Center, No. 651, Dongfeng East Road, 510060, Guangzhou City, Guangdong Province, People's Republic of China; 5 Department of Pathology, Sun Yat-sen University Cancer Center, Guangzhou, 510060, People's Republic of China

**Keywords:** Esophageal surgery, Skp2, Statistics, Survival analysis

## Abstract

**Background:**

The correlation of S-phase kinase–associated protein 2 (Skp2) with metastasis and prognosis in esophageal squamous cell carcinoma (ESCC) is controversial. The purpose of this study was to explore whether there was a correlation between the expression of Skp2 evaluated by immunohistochemistry and the clinical outcome of patients with operable ESCC, and to further determine the possible mechanism of the impact of Skp2 on survival.

**Methods:**

Tissue microarrays that included 157 surgically resected ESCC specimens was successfully generated for immunohistochemical evaluation. The clinical/prognostic significance of Skp2 expression was analyzed. Kaplan-Meier analysis was used to compare the postoperative survival between groups. The prognostic impact of clinicopathologic variables and Skp2 expression was evaluated using a Cox proportional hazards model. A cell proliferation assay and a colony formation assay were performed in ESCC cell lines to determine the function of Skp2 on the progression of ESCC *in vitro*.

**Results:**

Skp2 expression correlated closely with the T category (*p* = 0.035) and the pathological tumor-node-metastasis (TNM) stage (*p* = 0.027). High expression of Skp2 was associated with poor overall survival in resectable ESCC (*p* = 0.01). The multivariate Cox regression analysis demonstrated that pathological T category, pathological N category, cell differentiation, and negative Skp2 expression were independent factors for better overall survival. *In vitro* assays of ESCC cell lines demonstrated that Skp2 promoted the proliferative and colony-forming capacity of ESCCs.

**Conclusions:**

Negative Skp2 expression in primary resected ESCC is an independent factor for better survival. Skp2 may play a pro-proliferative role in ESCC cells.

## Background

Squamous cell carcinoma is the most common pathological esophageal cancer in the East [1], and surgery is still the best curative treatment option [2]. However, despite having comprehensive staging tests performed to determine which patients are most likely to benefit from potentially curative surgery, many patients develop tumor recurrence after surgery, and the 5-year survival rate is only approximately 42% [3]. The American Joint Committee on Cancer (AJCC) staging system [4] is commonly used in the assessment of clinical outcomes in patients with esophageal squamous cell carcinoma (ESCC). However, the system only reflects cancer progression status at the time of diagnosis, and some patients may survive for a long time without recurrence, whereas others with disease in the same tumor-node-metastasis (TNM) stage may have a more unfavorable prognosis due to tumor recurrence and metastasis. These differences are likely attributable to the inherent heterogeneity in the biological behavior of the tumors. Nevertheless, in view of the complexity of cancer progression [5-7], the availability of reliable markers is substantially limited. Thus, the combination of TNM staging with molecular markers and other clinicopathological parameters may be a promising method of selecting the patients with a high risk of postoperative recurrence, for the purpose of guiding tailored therapy.

Cell cycle regulation is critical for cell proliferation and tumorigenesis. One of the key players regulating cell cycle progression is the F-box protein, Skp2 (S-phase kinase–associated protein 2) [8,9]. Recent studies reveal that the overexpression of Skp2 is associated with the progression of a variety of human cancers [10-14]. However, the correlation of Skp2 expression with metastasis and prognosis in ESCC is still controversial [15,16].

In this study, we first evaluated Skp2 expression in resected ESCC by immunohistochemistry (IHC) using tissue microarrays. The correlation between Skp2 expression and clinicopathological characteristics and survival outcome was determined. We then used ESCC cell lines to further determine the function of Skp2 in the progression of ESCC.

## Methods

### Patient selection

This study was approved by the medical ethics committee of Sun Yat-sen University Cancer Center. A total of 157 primary ESCC patients who underwent complete resection at the Department of Thoracic Surgery at Sun Yat-sen University Cancer Center between December 1996 and October 2004 were eligible for inclusion in the study. All of the patients included in the study received complete resection and were re-staged according to the seventh edition of the AJCC Cancer Staging Manual [4]. We applied the definition of complete surgical resection proposed by the International Association for the Study of Lung Cancer (IASLC) Staging Committee [17].

All patients included in the analysis fit the following criteria: (1) their disease was histologically defined as thoracic squamous cell carcinoma; (2) they underwent complete resection; (3) they had complete information for stage grouping; (4) they fit into pathological AJCC stages I-III; (5) their resections were neither preceded nor followed by adjuvant chemotherapy or radiotherapy (esophagectomy alone); and (6) they had adequate paraffin-embedded cancer tissue samples for use in constructing the tissue microarray.

We excluded the patients with a history of concurrent malignant disease or other previous primary cancers and operative deaths. Operative death is defined as death within 30 days of the operation or any time after the operation if the patient did not leave the hospital alive.

### Follow-up of patients

The details of the strategy for patients’ follow-up in the training cohort were described in our previous study [18].

April 2009 was the last time of contact for the patients. The median time from surgery to the last time of contact for the training cohort was 90.2 months, ranging from 60.7 to 156.1 months.

### Tissue microarray construction

For uniform and simultaneous protein expression analysis of multiple tissue samples, we prepared tissue microarrays for IHC staining. Tumor tissue samples were collected, fixed in ethanol, and embedded in paraffin. H&E-stained sections from a single random block from each patient were reviewed by a senior pathologist to define representative tumor regions. For each sample, three tissue cylinders were taken from different representative tumor regions. Tissue cylinders with a diameter of 1 mm were punched from selected areas of each “donor” block using the Manual Tissue Arrayer (Beecher Instruments, Silver Spring, MD, USA) and arrayed into a recipient paraffin block. Serial 4 μm sections were mounted on silane-coated slides for IHC staining. The final IHC staining analyses included cores from 157 ESCC cases.

### IHC staining and scoring

IHC staining was performed using tissue microarray sections. The slides were deparaffinized in xylene, rehydrated through graded alcohols to water, heated in boiled EDTA buffer (pH 8.0) for 10 min, and cooled naturally to room temperature. They were subsequently immersed in 3% hydrogen peroxide solution for 10 min to remove endogenous peroxidase activity, blocked with 10% goat serum at room temperature for 30 min, and then incubated with mouse anti-Skp2 (1:40, Invitrogen) overnight (about 16 h) at 4°C. After being washed with PBS, the slides were incubated with secondary antibody (two-step anti-rabbit/mouse universal immunohistochemistry kit, Dako) at room temperature for 30 min and then developed with DAB for 6.5 min. Slides were counterstained with hematoxylin.

Two independent observers who were blinded to the clinicopathological information determined the immunoreactivity score (IRS) for each immunomarker. The staining results were scored based on the following criteria: (a) percentage of positive tumor cells in the tumor tissue: 0 (0%), 1 (1%–10%), 2 (11%–25%), 3 (26%–50%), 4 (51%–75%), or 5 (76%–100%); and (b) signal intensity: 0 (no staining), 1 (weak staining), 2 (moderate staining), or 3 (strong staining) [10]. IRS was calculated by multiplying the score for the percentage of positive cells by the intensity score (range of 0 to 15) [19]. The specimens were rescored if the difference between the scores determined by the two pathologists was greater than three [20]. If the conclusion was still controversial after the specimens were rescored, a third pathologist then intervened and worked collaboratively to find a consensus. The average IRS of each core determined by the two pathologists was assigned as the staining result for the core, and the average IRS of samples for each case was assigned as the staining result for the patient. The patients whose staining IRS were larger than zero were considered as positive expression of Skp2.

### Cell culture

ESCC cell lines KYSE30, KYSE140, and KYSE180 were kindly provided by Prof. Xin-Yuan Guan (Sun Yat-sen University Cancer Center). Cells were cultured in DMEM containing 10% fetal bovine serum at 37°C in a humidified atmosphere containing 5% CO_2_.

### Plasmids and siRNA transfection

Transient transfection of 50 nM of Skp2 small interfering RNA (siRNA) or scrambled siRNA was performed using Lipofectamine 2000 (Invitrogen) according to the manufacturer’s instructions. The sequences of the Skp2 siRNA, which were synthesized by the Ribobio Company (Guangzhou, China) as well as the scrambled siRNA (negative control, NC), were 5’-GGUAUCGCCUAGCGUCUGAdTdT-3’ for the sense primer and 5’-UCAGACGCUAGGCGAUACCdTdT-3’ for the anti-sense primer. The sense sequence of the scrambled siRNA was 5'-UUC UCC GAA CGU GUC ACG UTT-3', of which the anti-sense sequence was 5'-ACG UGA CAC GUU CGG AGA ATT-3'. The plasmids of pcDNA3.1-Skp2 and pcDNA3.1 empty vector were constructed in our laboratory and transiently transfected into ESCC cell lines using Lipofectamine 2000 (Invitrogen) according to the manufacturer’s instructions [21].

### Western blot assay

Cells were harvested 24 h after plasmid/siRNA transfection and lysed in a lysis buffer containing a cocktail of protease inhibitors. After centrifugation at 15,000 × g for 15 min at 4°C, supernatants were collected, mixed with dithiothreitol and used for western blotting. Equal amounts of protein extract were electrophoresed and then transferred to nitrocellulose membranes. The membranes were blocked with 3% non-fat milk at room temperature for 1 h, incubated with anti-Skp2 (1:1000, Invitrogen) overnight at 4°C, and incubated with the secondary antibody at room temperature for 1 h. After washing the blots, proteins were visualized by chemiluminescence.

### Cell proliferation assay

Cell proliferation was measured by a 3-[4, 5-dimethylthiazol-2-thiazolyl]-2,5- diphenyltetrazolium bromide (MTT) assay. ESCC cells were plated in 96-well plates at a density of 15,000-25,000 cells/mL in triplicate. Four hours before the desired time points, 20μL of 10 mg/mL MTT was added. After incubation for 4 h, the plates were depleted and 150 μL DMSO was added. The optical density (OD) values were determined by spectrophotometry (SpectraMax M5, Molecular Devices).

### Colony formation assay

Briefly, 24 h after plasmid/siRNA transfection, cells were trypsinized, resuspended as single cells, and plated in 6-well plates with 100 cells per well. After 14 days, the colonies were fixed with methanol and stained with crystal violet. Colonies with more than 50 cells were counted under the microscope. The cloning efficiency was calculated using the following formula: colony formation efficiency (%) = (clone number/inoculation cell number) × 100%.

### Statistical analysis

The SPSS statistical software package (Standard version 16.0; USA, Chicago, IL) was used for data analysis. The mean values are presented as the mean ± standard deviation (SD). Independent t-tests were used to compare groups of continuous, normally distributed variables. The Pearson chi-square test was used to determine the significant difference between groups for dichotomous variables. All statistical tests were two-tailed, and *p* < 0.05 was considered statistically significant. Survival time was measured from the date of surgery to the date of event or last follow-up. Patients alive at the end of the study were censored for the purpose of data analysis. Multivariate survival analysis was performed using the Cox regression model.

## Results

### General patient characteristics

A total of 157 cases fit the inclusion criteria and were included in this study. Table 1 provides the details of the clinicopathological features of the entire cohort of patients.

**Table 1:** Skp2 expression and Clinicopathological characteristics for entire cohort of patients

**Characteristics**	**Case**				**Skp2 Expression (%)**
		**Negative**	**Positive**	***p***	
Age(years)				
≤60	96	50 (52.1)	46 (47.9)	0.144
>60	61	39 (63.9)	22 (36.1)	
Gender				
Male	126	69 (54.8)	57 (45.2)	0.326
Female	31	20 (64.5)	11 (35.5)	
Tumor location				
Upper	20	12 (60.0)	8 (40.0)	0.796
Middle	76	41 (53.9)	35 (46.1)	
Lower	61	36 (59.0)	25 (41.0)	
Cell differentiation				
G1	27	15 (55.6)	12 (44.4)	0.861
G2	80	47 (58.8)	33 (41.2)	
G3	50	27 (54.0)	23 (46.0)	
Pathological T status				
T1	15	11 (73.3)	4 (26.7)	0.035
T2	42	29 (69.0)	13 (31.0)	
*T3-4	100	49 (49.0)	51 (51.0)	
Pathological N status				
N0	87	52 (59.8)	35 (40.2)	0.438
N1	46	27 (58.7)	19 (41.3)	
N2	21	9 (42.9)	12 (57.1)	
N3	3	1 (33.3)	2 (66.7)	
AJCC stage				
I	37	28 (75.7)	9 (24.3)	0.027
II	65	34 (52.3)	31 (47.7)	
III	55	27 (49.1)	28 (50.9)	
Overall	157	89 (56.7)	68 (43.3)	—

G1, well differentiated; G2, moderately differentiated; G3, poorly differentiated; AJCC, American Joint Committee on Cancer; *T3: 99 cases, T4: 1 case with negative Skp2 expression.

### Expression of Skp2 in ESCC

**Figure 1:**
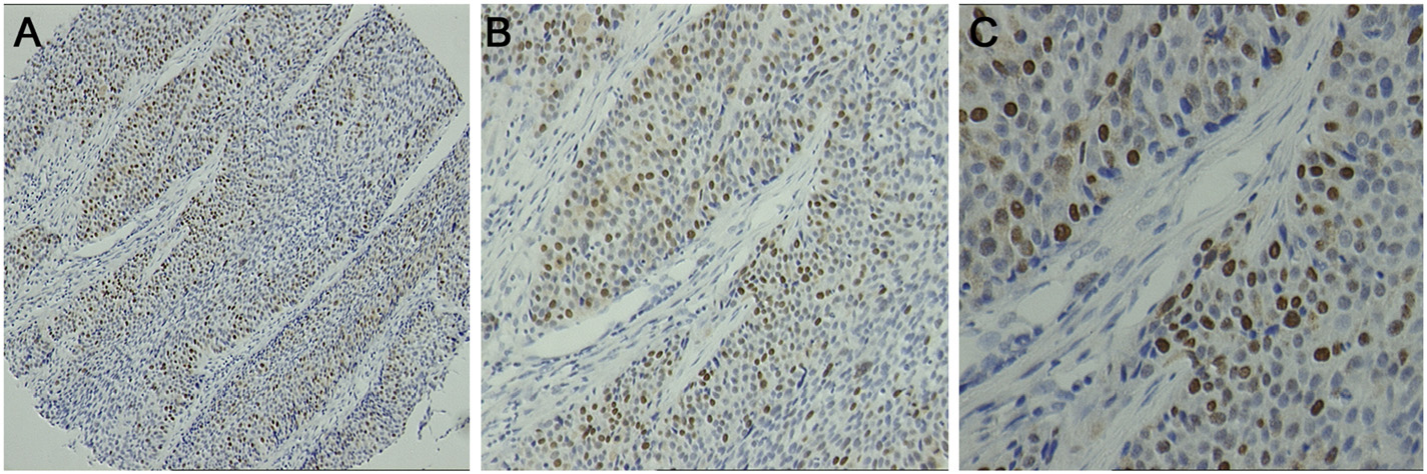
**Photographs of tissue sections immunostained for Skp2, showing that Skp2 was detected in the nuclei of the esophageal squamous carcinoma cells**. (**A**): x100; (**B**): x200; (**C**): x400.

In the present study, Skp2 staining of ESCC tissue revealed immunoreactivity primarily in nuclei within tumor cells (Figure 1). Positive Skp2 expression was observed in 43.3% (68/157) of ESCC cases. No statistically significant correlation was observed between Skp2 expression and age, sex, tumor location, histological grade, or N categories (Table 1), but the Skp2 expression correlated closely with the T category (*p* = 0.035) and the pathological AJCC stage (*p* = 0.027).

### Survival analysis

**Figure 2:**
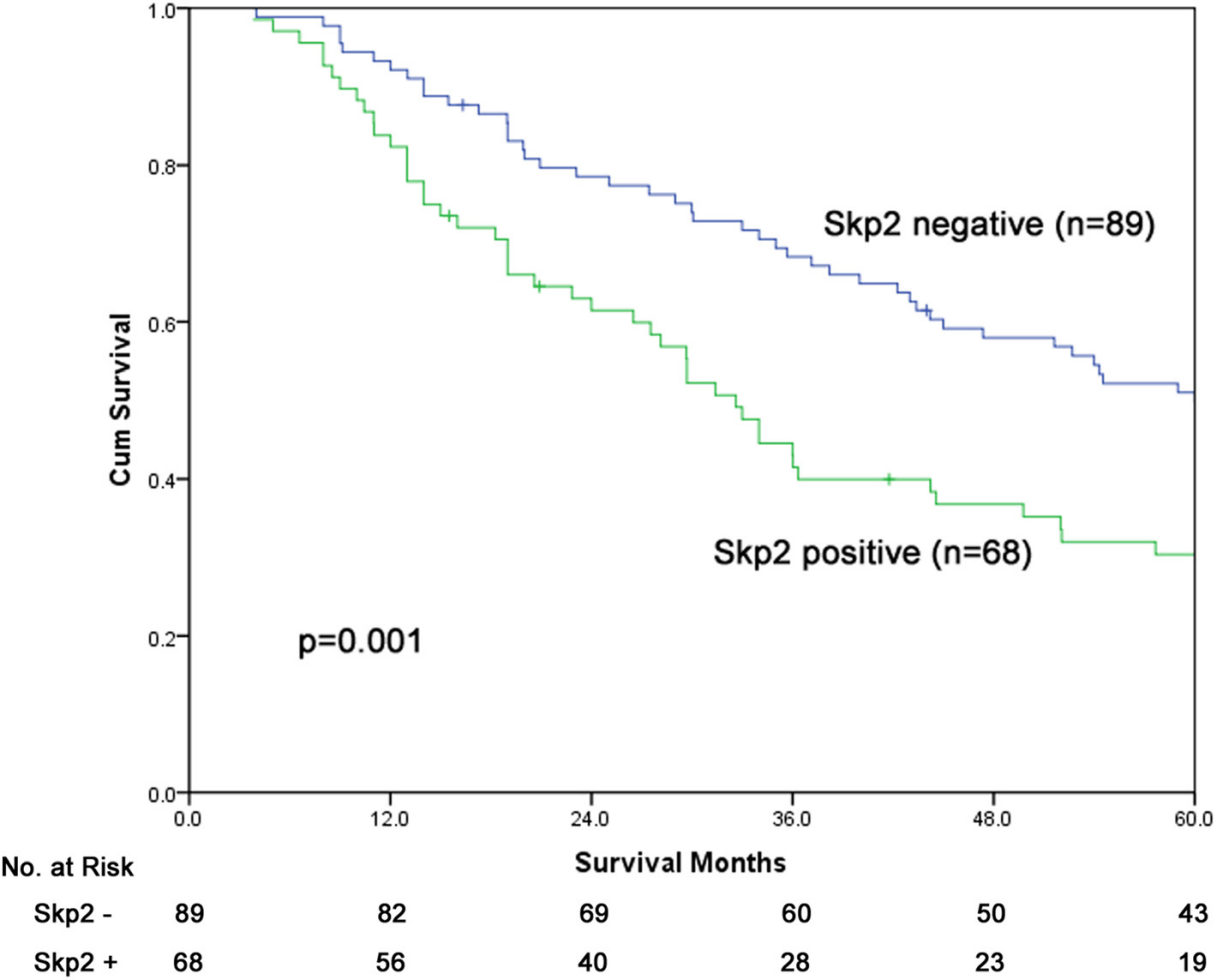
Overall survival curves for patients with positive and negative expression of Skp2.

The 2-year, 3-year, and 5-year survival rates for the entire cohort of 157 cases were 71.2%, 57.4%, and 41.5%, respectively, with a median survival of 45.0 months. In patients with negative Skp2 expression, the 2-year, 3-year, and 5-year survival rates were 78.5%, 68.3%, and 49.9%, respectively, with a median survival of 60.0 months. In patients with positive Skp2 expression, the 2-year, 3-year, and 5-year survival rates were 61.5%, 43.0%, and 30.4%, respectively, with a median survival of 32.6 months. Patients with positive Skp2 expression exhibited decreased survival time compared with those with negative expression (*p* = 0.001, Figure 2).

Multivariate Cox regression analysis showed that Skp2 expression, pathological T category, pathological N category, and cell differentiation were independent factors of overall survival (Table 2).

**Table 2:** Results of multivariate analyses of overall survival by Cox regression model

**Variables**	**RR**			**95% CI**	***p***
		**Negative**	**Positive**		
Age (≤60 vs. > 60)	0.950	0.639	1.413	0.799
Gender (male vs. female)	1.592	0.924	2.741	0.094
Cell differentiation (G1-2 vs. G3)	0.594	0.395	0.892	0.012
T categories (T1-2 vs. T3-4)	0.612	0.390	0.961	0.033
N categories (N0 vs.N1-3)	0.357	0.237	0.537	<0.01
Skp2 expression (negative vs. positive)	0.627	0.418	0.941	0.024

RR, relative risk; CI, confidence interval, G1, well differentiated; G2, moderately differentiated; G3, poorly differentiated.

### The effect of Skp2 on cell proliferation in ESCC cells

**Figure 3:**
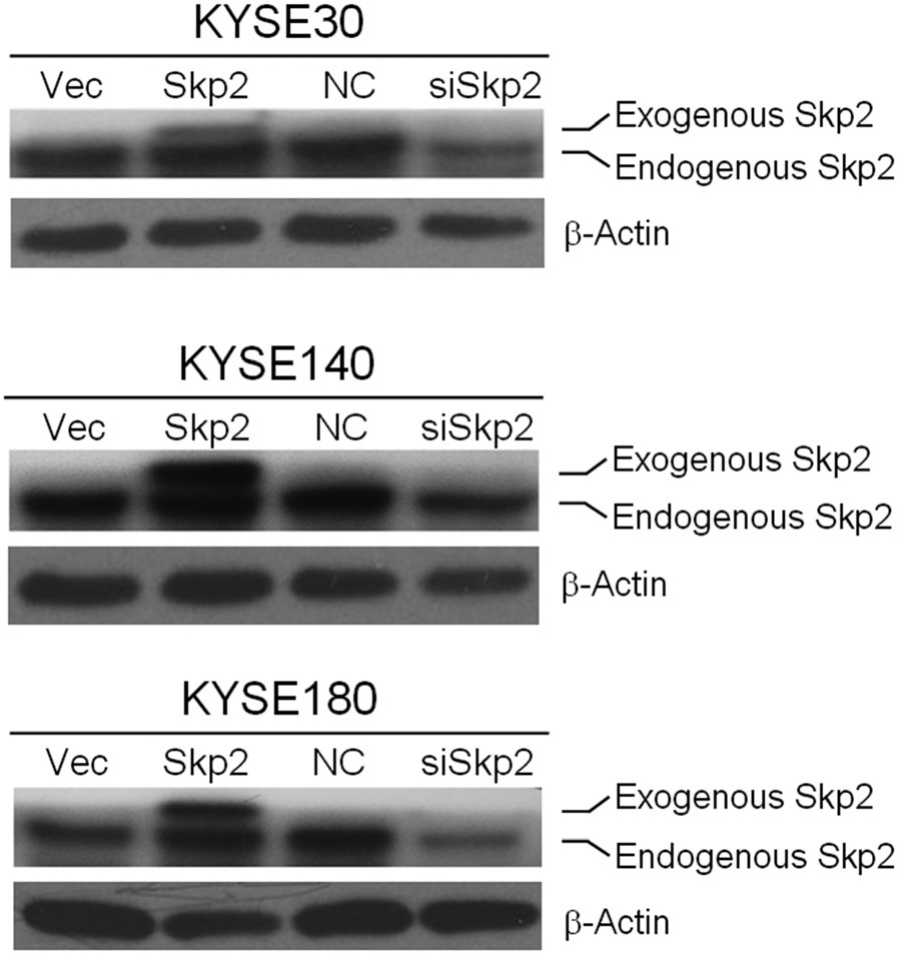
**Transient overexpression or knockdown of Skp2 in ESCC cell lines**. A western blot assay was used to detect the protein level of Skp2 36 h after transient transfection of an empty pcDNA3.1 vector (Vec), pcDNA3.1-Skp2 plasmid (Skp2), scrambled siRNA as negative control (NC) or the siRNA specifically targeting Skp2 (siSkp2). (**A**) KYSE30 cell line; (**B**) KYSE140 cell line; (**C**) KYSE180 cell line. The exogenous/endogenous Skp2 is indicated in the figure. β-Actin was used as a loading control.

**Figure 4:**
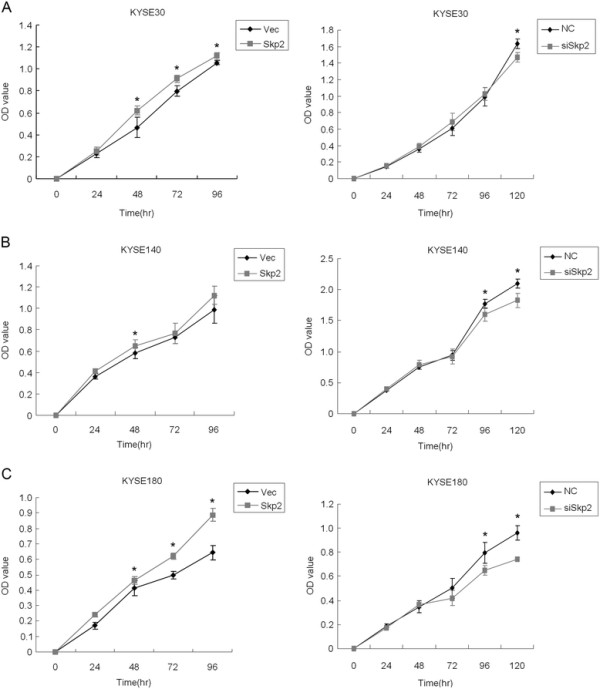
**Cell proliferation of ESCC cell lines after overexpression or knockdown of Skp2**. MTT assays were used to measure the optical density (OD) values of cells transfected by an empty pcDNA3.1 vector (Vec), pcDNA3.1-Skp2 plasmid (Skp2), scrambled siRNA as negative control (NC) or the siRNA specifically targeting Skp2 (siSkp2) at the indicated time points. (**A**) In KYSE30 cell line. For left panel, the asterisks represent as *p* = 0.004 (48 h), 0.001 (72 h), and 0.002 (96 h), respectively. For right panel, the asterisk represents as *p* = 0.001 (120 h). (**B**) In KYSE140 cell line. For left panel, the asterisk represents as *p* = 0.024 (48 h). For right panel, the asterisks represent as *p* = 0.016 (96 h) and 0.003 (120 h), respectively. (**C**) In KYSE180 cell line. For left panel, the asterisks represent as *p* = 0.042 (48 h), *p* < 0.001 (72 h), and *p* < 0.001 (96 h), respectively. For right panel, the asterisks represent as *p* = 0.004 (96 h) and *p* < 0.001 (120 h), respectively. Data are represented as mean +/- SD.

Because the expression of Skp2 significantly correlated with the T category and overall survival, we proposed that Skp2 could play a functional role in the cell proliferation of ESCC. Hence, we performed a MTT assay to assess the effect of Skp2 on cell proliferation in ESCC cell lines, including KYSE30, KYSE140 and KYSE180. By transient transfection of the pcDNA3.1-Skp2 plasmid, we observed the overexpression of Skp2 in those cell lines by Western Blot assays (Figure 3). Interestingly, enhanced expression of Skp2 remarkably promoted the proliferation of ESCC cells (the left panels of Figure 4A-C). In contrast, we utilized siRNA specifically targeting Skp2 to knockdown the protein level of Skp2 (Figure 3) and found that decreased expression of Skp2 attenuated the proliferation of ESCC cells (the right panels of Figure 4A-C). In summary, those observations demonstrated the functional role of Skp2 in promoting the proliferation of ESCC cells.

### The effect of Skp2 on colony formation in ESCC cells

**Figure 5:**
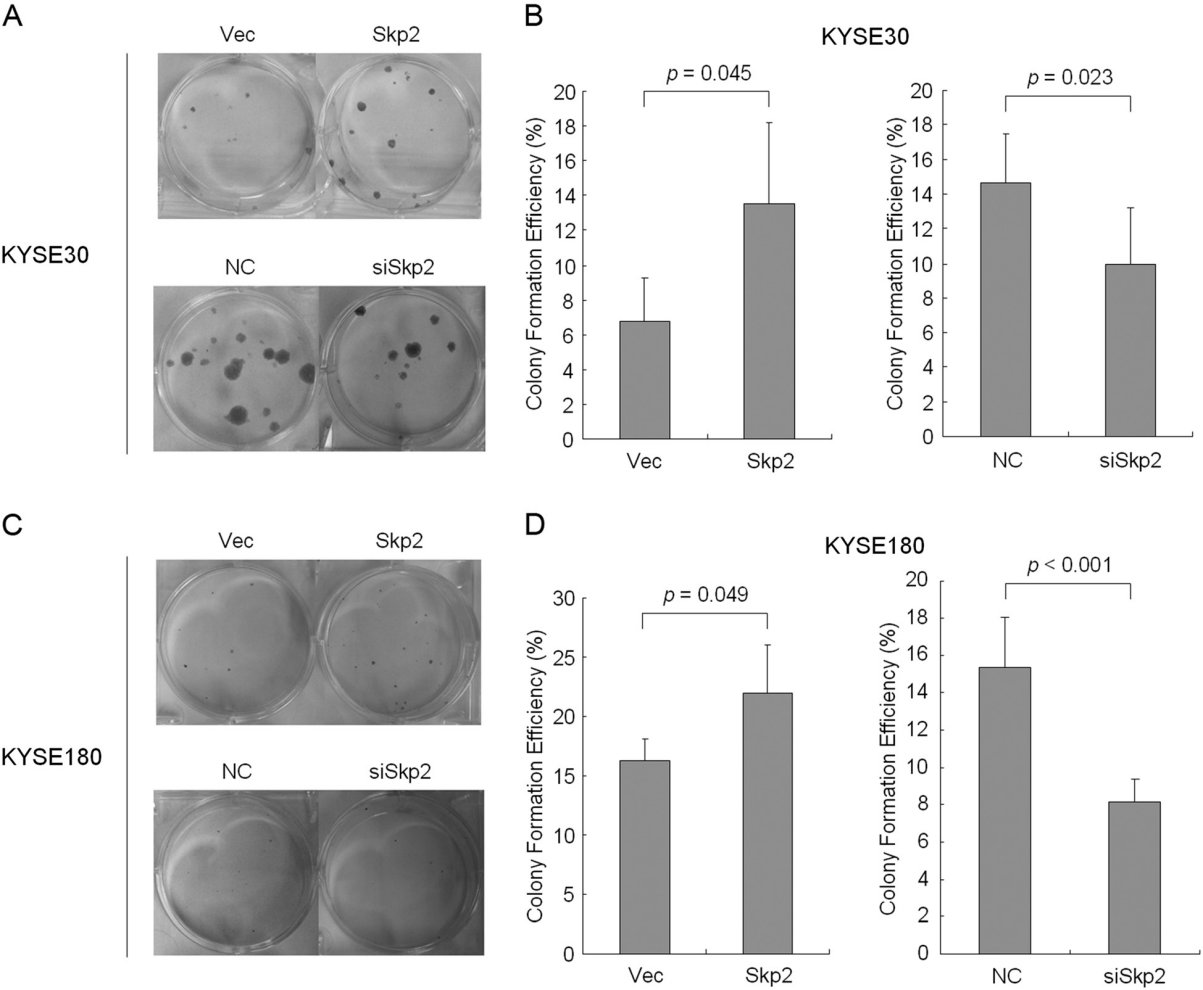
**Colony formation results of ESCC cell lines after overexpression or knockdown of Skp2**. Colonies were counted 2 ~ 3 weeks after cells transfected by an empty pcDNA3.1 vector (Vec), pcDNA3.1-Skp2 plasmid (Skp2), scrambled siRNA as negative control (NC) or the siRNA specifically targeting Skp2 (siSkp2) were planted in 6-well plates. (**A**) Representative photographs of colony formation in KYSE30 cell line; (**B**) Statistical analysis in KYSE30 cell line. (**C**) Representative photographs of colony formation in KYSE180 cell line; (**D**) Statistical analysis in KYSE180 cell line. Data are represented as mean +/- SD. The *p* values are indicated in the figure.

In addition to cell proliferation assays, we performed colony formation experiments to further explore the function of Skp2 in ESCC cells. Except that KYSE140 cells seldom or never formed colonies in plates (data not shown), in both KYSE30 and KYSE180 cell lines, overexpression of Skp2 could dramatically enhance the potential of colony formation (the upper panels of Figure 5A and 5C, see also the left panels of Figure 5B and 5D). Conversely, knockdown of Skp2 led to a significant decrease in colony-forming efficiency (the lower panels of Figure 5A and 5C, see also the right panels of Figure 5B-5D). Therefore, our *in vitro* experimental results documented that Skp2 could not only promote cell proliferation but also facilitate colony formation in ESCC cells.

### The anti-proliferative effect of proteasome inhibitor in ESCC cells

**Figure 6:**
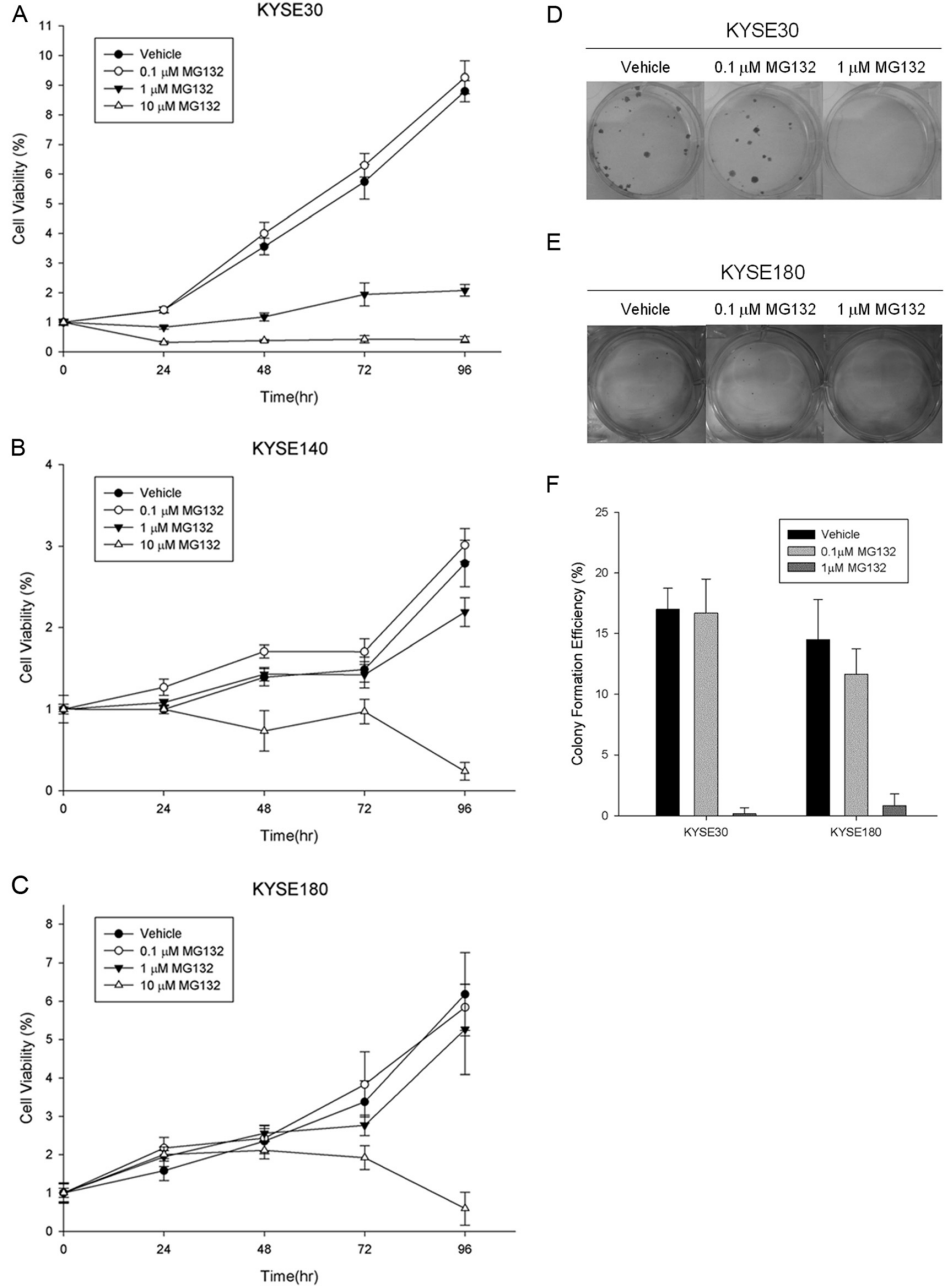
**The inhibitive effect of proteasome inhibitor MG132 on cell proliferation and colony formation in ESCC cells**. MTT assays were used to assess the cell viability of cells constantly treated by MG132 at the indicated time points. The concentrations of MG132 are indicated in the figure. (**A**) KYSE30 cell line; (**B**) KYSE140 cell line; (**C**) KYSE180 cell line. Colonies were counted 2 ~ 3 weeks after cells were seeded and constantly treated by MG132 at the indicated concentrations. (**D**) Representative photographs of colony formation in KYSE30 cell line; (**E**) Representative photographs of colony formation in KYSE180 cell line; (**F**) Statistical analysis in KYSE30 and KYSE180 cell lines. Data are represented as mean +/- SD.

Based on the essential role of Skp2 in the participation of the protein ubiqutination, we further investigated the effect of proteasome inhibitor on cell proliferation and colony formation in ESCC cells. We tested the cell proliferation and colony formation with/without MG132, one commonly used proteasome inhibitor, in ESCC cell lines KYSE30, KYSE140 and KYSE180. With the constant treatment of MG132 at the concentration of 10 μM, the proliferation of all the three cell lines was significantly aborted. However, KYSE30 cells could be highly eliminated with the treatment of 1 μM MG132 as well, showing much higher sensitivity to the inhibition of MG132 than KYSE140 and KYSE180 cells (Figure 6A-6C). In the colony formation assays, both KYSE30 and KYSE180 cells were totally abolished to produce colonies in the presence of 1 μM MG132 (Figure 6D-6F). Taken together, our data indicated that inhibition of protein ubiqutination could depress the cell proliferation and colony formation in ESCC cells, suggesting the potential application of proteasome inhibitor in the therapy for human ESCC.

## Discussion

Skp2 was originally identified as an S-phase Kinase Cdk2/Cyclin A-associated protein [22]. Skp2 is known as a critical component of the Skp2/SCF (Skp1-Cullin1-F-box) complex, which is capable of inducing protein ubiquitination and subsequent proteasome-dependent degradation [23,24]. Among the numerous substrates for degradation, many are negative cell cycle regulators, including p27^Kip1^, p21^Cip1^ and p57^Kip2^[25,26]. The ubiquitylated p27^Kip1^ is then rapidly destroyed by the proteasome, allowing the activity of cyclin E/A-CDK2 and progression to the S phase [27]. Overexpression of Skp2 significantly promotes the transition from G1 phase to S phase, therefore leading to the accelerated proliferation. In addition, Skp2 may contribute to the resistance to apoptosis induction by DNA damage agents, such as irradiation and doxorubicin [28].

Previous studies demonstrated that Skp2 was commonly overexpressed and associated with poor prognosis in a variety of human cancers, including gastrointestinal cancer [27-29], breast cancer [30,31], prostate cancer [32,33], lung cancer [34,35] and nasopharyngeal cancer [14,21]. However, the prognostic role of Skp2 in ESCC has been seldom investigated and is still controversial. Fukuchi et al. determined the Skp2 expression in a small number (32 cases) of ESCC, in which they suggested that Skp2 might be a prognostic factor in early stage ESCC [15]. Recently, Wang et al. reported that overexpression of Skp2 correlated with lymph node metastasis and advanced tumor stage in ESCC (140 cases), but unfortunately, the survival prognosis was not determined in their study [16].

In the present study, we initially showed that high expression of Skp2 in tumor cell nuclei was associated with advanced local invasion of primary carcinoma. Moreover, the positive expression of Skp2 in this study is indicative of unfavorable prognosis. Multivariate Cox proportional hazards analysis further confirmed that high Skp2 expression was an unfavorable prognostic factor independent of conventional prognostic factors, such as T categories and N categories. These results indicated that Skp2 could influence tumor progression of ESCC, and Skp2 could be useful in predicting tumor recurrence for operable ESCC. Our data showed that Skp2 expression did not correlate with lymph node metastasis, but correlated with local tumor invasion. However, Wang et al. reported high expression of Skp2 associated with lymph node metastasis, but not with local tumor invasion [16], which was contrary to that of our study. The controversy about Skp2 expression and tumor progression also exists in colorectal carcinoma [10,27]. These controversies suggest that more studies are warranted for further assessment of the role of Skp2 in cancer progression. A possible mechanism for these controversies is the heterogeneity of cancer. Skp2 is an essential regulator of the cell cycle, probably playing multiple roles in carcinogenesis. In the present study, the case number is much larger than in previous studies [15,16]. The large number and multivariate Cox survival analysis make our outcome much more reliable.

Our data from *in vitro* experiments, including cell proliferation and colony formation, provided evidence for Skp2’s role in facilitating cancer progression, consistent with the survival analysis results of ESCC patients. In contrast to Wang et al.’s observation that Skp2 may promote ESCC metastasis via inhibiting anoikis [16], our findings exhibit a possibility that Skp2 directly speeds up the proliferation of ESCC cells. If this is the case, our discovery that Skp2 expression correlates with T stage but not N stage can be explained. Based on this discovery, we did not perform experiments to test the mobility of ESCC cells in which Skp2 was overexpressed or knocked down, since we did not found a correlation between the level of Skp2 expression and lymph node metastasis in our clinicopathological data.

Our data suggests that Skp2 expression might be a new prognostic biomarker for tumor recurrence in ESCC patients. Therefore, the examination of Skp2 expression, detected by IHC, could be used as an additional tool for identifying those patients who are more likely to suffer from recurrence after surgery. Thus, designing an optimized individual adjuvant therapy strategy for resectable ESCC patients will become possible. According to the practice guidelines of the National Comprehensive Cancer Network (NCCN), adjuvant chemotherapy was not recommended for completely resected ESCC [36]. However, many patients develop local recurrence or metastasis after surgery, and the 5-year survival rate is only about 42% [3]. For application purposes, if a patient with operable ESCC is with high expression of Skp2, adjuvant chemotherapy can be recommended after surgery; on the contrary, for patients with a low expression of Skp2, observation is preferred. Although further evaluation of this strategy for clinical use will be necessary, it may help clinicians to select the most appropriate therapies for individual ESCC patients in advance.

Despite the finding that Skp2 expression affects the survival of patients with ESCC and the proliferation of ESCC cells, our study has its limitations. On one hand, there is a lack of investigation on the molecular mechanism for the proliferative promotion of Skp2 in ESCC cells. It still remains unclear which signal pathway is involved in the Skp2-dependent regulation of ESCC proliferation. On the other hand, more experiments, such as a tumorigenesis assay in a xenograft model *in vivo*, are needed to further validate the oncogenic function of Skp2 in ESCC.

## Conclusions

Skp2 expression, as determined by IHC in operable ESCC, is a potential prognostic biomarker. The combination of AJCC staging with Skp2 expression may be useful in identifying patients with increased risk of cancer recurrence for complete resected ESCC. Furthermore, Skp2 may play a pro-proliferative role in ESCC cells. As a consequence, the presence of a Skp2 inhibitor might advance ESCC therapeutic strategy. Further studies are required to validate our results.

## List of abbreviations

AJCC = The American Joint Committee on Cancer; ESCC = esophageal squamous cell carcinoma; TNM = tumor-node-metastasis; Skp2 = S-phase kinase–associated protein 2; IHC = immunohistochemistry; CT = computerized tomography; IRS = immunoreactivity score; MTT = 3-[4,5-dimethylthiazol-2-thiazolyl]-2,5- diphenyltetrazolium bromide; OD = optical density; SD = standard deviation; NCCN = the National Comprehensive Cancer Network.

## Misc

Yi Liang, Xue Hou contributed equally to this work and share the first authorship.

## Competing interests

The authors declare that they have no competing interests.

## Authors' contributions

YL, XH and HXY conceived the study, designed, performed and analyzed all experiments and wrote the manuscript. Q. Cui participated in Western blot assays. RZL and JHH participated in IHC studies. TBK, JHF, LJZ and YXZ participated in conceiving the study. All authors read and approved the final version of the manuscript.
